# Preoperative pulmonary function correlates with systemic inflammatory response and prognosis in patients with non-small cell lung cancer: results of a single-institution retrospective study

**DOI:** 10.18632/oncotarget.14225

**Published:** 2016-12-25

**Authors:** Yongyin Gao, Hongdian Zhang, Yue Li, Dandan Wang, Yinlu Ma, Qing Chen

**Affiliations:** ^1^ Department of Cardio-pulmonary Functions, Tianjin Medical University Cancer Institute and Hospital, National Clinical Research Center for Cancer, Key Laboratory of Cancer Prevention and therapy, Tianjin, China; ^2^ Department of Esophageal Cancer, Tianjin Medical University Cancer Institute and Hospital, National Clinical Research Center for Cancer, Key Laboratory of Cancer Prevention and therapy, Tianjin, China

**Keywords:** non-small cell lung cancer, pulmonary function, systemic inflammatory response, prognosis

## Abstract

This study aimed at analyzing the relationship between preoperative pulmonary function and systemic inflammatory response (SIR) biomarkers, such as neutrophil-to-lymphocyte ratio (NLR), platelet-to-lymphocyte ratio (PLR) and lymphocyte-to-monocyte ratio (LMR) in patients with non-small cell lung cancer (NSCLC). Furthermore, the prognostic significance of these markers was also examined. The medical records of 358 NSCLC patients, who underwent curative lung resection, were retrospectively analyzed. Pulmonary function test values <80% of the predicted values were used to indicate impairment. A receiver operating characteristic curve was used to determine the thresholds of the SIR biomarkers. Univariate and multivariate survival analyses were then performed to identify the factors associated with the overall survival (OS). Furthermore, one prognostic model based on independent prognostic factors was established to classify the patients into low-, intermediate-, and high-risk groups. Results demonstrated that, preoperative forced vital capacity (FVC) was simultaneously associated with NLR, PLR, and LMR (*P* < 0.05). Multivariate analysis identified age, lymph node status, FVC, and NLR as independent prognostic factors for OS. A subgroup analysis showed that the prognostic value of FVC was independent of age, lymph node status, and NLR. The five-year OS rates for low-, intermediate-, and high-risk groups of prognostic model were 60.9%, 35.9%, and 15.3%, respectively (*P* < 0.05). Overall, preoperative FVC was an independent prognostic predictor of NSCLC. Significant correlations were observed among preoperative pulmonary function, SIR, and prognosis. Thus, the prognostic model may help us identify risk populations with NSCLC.

## INTRODUCTION

Lung cancer remains the most common form of cancer and the leading cause of cancer-related deaths worldwide. Non-small cell lung cancer (NSCLC) accounts for approximately 80% of all lung cancers [1]. Radical surgical resection offers the chance to achieve long-term survival and cure, and is considered as the optimal treatment for patients with operable NSCLC. However, many patients with lung cancer have respiratory deficiencies, impeding surgical resection [2].

Preoperative pulmonary function plays an important role in the evaluation of surgery tolerance, lung reserve, risk of perioperative complications, and short-term outcome [3, 4]. The forced expiratory volume in one second (FEV1) and diffusion lung capacity for carbon monoxide (DLCO) are useful parameters for predicting the perioperative complications and short-term mortality [5, 6] and long-term survival in NSCLC [2, 3]. However, results from previous studies have not yet reached a unified conclusion and are even controversial [4]. Forced vital capacity (FVC) is associated with cardiovascular disease mortality [4]. However, apart from FEV1 and DLCO, few studies have analyzed the effect of FVC and other pulmonary function parameters on the long-term survival of NSCLC patients.

Increasing data have indicated that the prognosis of cancer patients was based not only on tumor-related factors but also on host-related factors, particularly the systemic inflammatory response (SIR). SIR promotes tumor angiogenesis, accelerates tumor cells metastasis, and suppresses antitumor immunity [7]. Mounting evidence supports the prognostic role of systemic inflammatory laboratory parameters, such as neutrophil-to-lymphocyte ratio (NLR), platelet-to-lymphocyte ratio (PLR), and lymphocyte-to-monocyte ratio (LMR), in patients with NSCLC undergoing surgery resection [8–10]. Furthermore, previous reports have revealed that elevated SIR may insult the pulmonary function and increase the peripheral serum white blood cell count, mononuclear cells and lymphocytes were associated with impaired pulmonary function [11, 12]. However, few studies have evaluated the relationship between pulmonary function and SIR biomarkers (NLR, PLR, and LMR) in patients with NSCLC. Thus, considering the available data, we attempted to determine whether preoperative pulmonary functions are associated with SIR biomarkers in patients with NSCLC.

Therefore, in the present study, we determined the optimum preoperative pulmonary function parameters associated with long-term survival in patients with NCSLC by including as many pulmonary function parameters as possible. Furthermore, we investigated for the first time the associations among preoperative pulmonary function, SIR biomarkers, and prognosis in NSCLC.

## RESULTS

### Clinicopathological characteristics of patients

The baseline clinicopathological characteristics of 358 patients with primary and histologically confirmed NSCLC were summarized in Table 1. There were 247 (69.0%) males and 111 (31.0%) females with the median age of 61 years (range: 25∼83 years). Meanwhile, 244 (68.2%) patients had smoking history. After complete resection, 125 (34.9%) patients had adenocarcinoma, 162 (45.3%) patients had squamous cell carcinoma, and 71 (19.8%) patients were not specified. Approximately 38.8% of the patients had lymph node metastasis. The distribution of TNM stages was as follows: stage I, 173 patients; stage II, 87 patients; and stage III, 98 patients. Furthermore, 166 (46.4%) patients had FVC% predicted of < 80%, and 213 (59.5%) patients had FEV1% predicted of < 80%. Impaired vital capacity (VC), FEV1/FVC, peak expiratory flow (PEF), mid-expiratory flow when 50% of FVC has been expired (MEF50%), DLCO, and maximal voluntary ventilation (MVV) were observed in 196, 264, 280, 249, 118, and 123 patients, respectively.

**Table 1:** Demographics and characteristics of the study population of NSCLC individuals

Variables	Cases (%)
Age (years)	
≤ 60/ > 60	175 (48.9%)/ 183 (51.1%)
Gender	
Male/ Female	247 (69.0%)/ 111 (31.0%)
Smoking history	
Yes/ None	244 (68.2%)/ 114 (31.8%)
Type of resection	
Pneumonectomy/ Lobectomy/ bronchial sleeve resection	92 (25.7%)/ 249 (69.6%)/ 17 (4.7%)
Histology	
Adenocarcinoma/ Squamous cell carcinoma/ Others	125 (34.9%)/ 162 (45.3%)/ 71 (19.8%)
T stage	
T1/ T2/ T3-4	152 (42.5%)/ 185 (51.7%)/ 21 (5.9%)
Lymph node status	
N0/ N+	219 (61.2%)/ 139 (38.8%)
TNM stage	
I/ II/ III	173 (48.3%)/ 87 (24.3%)/ 98 (27.4%)
VC	
Normal/ Impairement	162 (45.3%)/ 196 (45.7%)
FVC	
Normal/ Impairement	192 (53.6%)/ 166 (46.4%)
FEV1	
Normal/ Impairement	145 (40.5%)/ 213(59.5%)
FEV1/FVC	
Normal/ Impairement	94 (26.3%)/ 264 (73.7%)
PEF	
Normal/ Impairement	78 (21.8%)/ 280 (78.2%)
MEF50%	
Normal/ Impairement	109 (30.4%)/ 249 (69.6%)
DLCO	
Normal/ Impairement	240 (67.0%)/ 118 (33.0%)
MVV	
Normal/ Impairement	235 (65.6%)/ 123 (34.4%)
NLR	
<2.14/ ≥2.14	196 (54.7%)/ 162 (45.3%)
PLR	
<121.78/ ≥121.78	179 (50.0%)/ 179 (50.0%)
LMR	
<3.06/ ≥3.06	127 (35.5%)/ 231 (64.5%)

### Cut-point survival analysis for the detection of the optimum cut-points of NLR, PLR, and LMR

**Figure 1:**
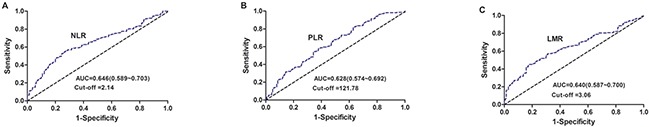
Receiver operating characteristic curve analysis for the cut-points of NLR, PLR, and LMR in NSCLC patients **A**. The AUC for NLR was 0.646 (95%CI: 0.589∼0.703) with a sensitivity of 55.5% and specificity of 72.9%. **B**. The AUC for PLR was 0.628 (95%CI: 0.574∼0.692) with a sensitivity of 57.8% and specificity of 62.1%. **C**. The AUC for LMR was 0.640 (95%CI: 0.587∼0.700) with a sensitivity of 45.0% and specificity of 82.1%.

The optimum cut-point values of the preoperative NLR, PLR, and LMR for survival prediction were determined through a receiver operating characteristic curve (ROC) curve analysis. For NLR, the optimum cut-point was 2.14 with a maximum joint sensitivity of 55.5% and specificity of 72.9% on the ROC plot. The area under the ROC curve (AUC) was 0.646 (95% CI: 0.589∼0.703). For PLR, the optimum cut-point was 121.78 with a maximum joint sensitivity of 57.8% and specificity of 62.1%, and the AUC was 0.628 (95%CI: 0.574∼0.692). For LMR, the optimum cut-point was 3.06 with a maximum joint sensitivity of 45.0% and specificity of 82.1%, and the AUC was 0.640 (95% CI: 0.587∼0.700) (Figure 1). Therefore, NLR, PLR, and LMR with cut-point values of 2.14, 121.78, and 3.06, respectively, were used as optimum cut-points in the subsequent analysis. For each biomarker, the patients were classified into two groups (NLR [<2.14 and ≥2.14], PLR [<121.78 and ≥121.78], and LMR [<3.06 and ≥3.06]).

### Correlation analysis between preoperative pulmonary function and SIR biomarkers

The correlations between preoperative pulmonary function and SIR biomarkers (NLR, PLR, LMR) were analyzed. As shown in Table 2, impaired VC was associated with elevated PLR (*x^2^* = 6.494, *P* = 0.011). Impaired FEV1 and PEF were associated with decreased LMR (*x^2^* = 4.551, *P* = 0.034; *x^2^* = 13.383, *P* <0.001). Lastly, impaired DLCO was associated with elevated NLR (*x^2^* = 4.706, *P* = 0.030) and PLR (*x^2^* = 5.056, *P* = 0.025). Notably, impaired FVC was simultaneously associated with elevated NLR (*x^2^* = 4.428, *P* = 0.035) and PLR (*x^2^* = 6.470, *P* = 0.011) and decreased LMR (*x^2^* = 4.074, *P* = 0.044). However, statistically significant association was not observed among other pulmonary function parameters (FEV1/FVC, MEF50%, MVV) and NLR, PLR, or LMR (*P* > 0.05).

**Table 2:** Correlation analysis of preoperative pulmonary function parameters and SIR biomarkers in NSCLC patients

Variables	N	NLR		Chi-square test		PLR		Chi-square test		LMR		Chi-square test	
		Low	High	x^2^	P	Low	High	x^2^	P	Low	High	x^2^	P
		196	162			179	179			127	231		
VC				2.430	0.119			6.494	**0.011**			2.748	0.097
Normal	162	96 (59.3%)	66 (40.7%)			93 (57.4%)	69 (42.6%)			50 (30.9%)	112 (69.1%)		
Impairement	196	100 (51.0%)	96 (49.0%)			86 (43.9%)	110 (56.1%)			77 (39.3%)	119 (60.7%)		
FVC				4.428	**0.035**			6.470	**0.011**			4.074	**0.044**
Normal	192	115 (59.9%)	77 (40.1%)			108 (56.3%)	84 (43.8%)			59 (30.7%)	133 (69.3%)		
Impairement	166	81 (48.8%)	85 (51.2%)			71 (42.8%)	95 (57.2%)			68 (41.0%)	98 (59.0%)		
FEV1				0.320	0.572			0.939	0.333			4.511	**0.034**
Normal	145	82 (56.6%)	63 (43.4%)			77 (53.1%)	68 (46.9%)			42 (29.0%)	103 (71.0%)		
Impairement	213	114 (53.5%)	99 (46.5%)			102 (47.9%)	111 (52.1%)			85 (39.9%)	128 (60.1%)		
FEV1/FVC				0.353	0.552			0.923	0.337			0.114	0.735
Normal	94	49 (52.1%)	45 (47.9%)			51 (54.3%)	43 (45.7%)			32 (34.0%)	62 (66.0%)		
Impairement	264	147 (55.7%)	117 (44.3%)			128 (48.5%)	136 (51.5%)			95 (36.0%)	169 (64.0%)		
PEF				0.093	0.760			0.013	0.909			13.383	**0.000**
Normal	109	61 (56.0%)	48 (44.0%)			54 (49.5%)	55 (50.5%)			14 (17.9%)	64 (82.1%)		
Impairement	249	135 (54.2%)	114 (45.8%)			125 (50.2%)	124 (49.8%)			113 (40.4%)	167 (59.6%)		
MEF 50%				0.093	0.760			0.646	0.412			3.388	0.066
Normal	109	61 (56.0%)	48 (44.0%)			58 (53.2%)	51 (46.8%)			31 (28.4%)	78 (71.6%)		
Impairement	249	135 (54.2%)	114 (45.8%)			121 (48.6%)	128 (51.4%)			96 (38.6%)	153 (61.4%)		
DLCO				4.706	**0.030**			5.056	**0.025**			0.946	0.331
Normal	240	141 (58.8%)	99 (41.3%)			130 (54.2%)	110 (45.8%)			81 (33.8%)	159 (66.3%)		
Impairement	118	55 (46.6%)	63 (53.4%)			49 (41.5%)	69 (58.5%)			46 (39.0%)	72 (61.0%)		
MVV				0.090	0.764			0.310	0.578			1.031	0.310
Normal	235	130 (55.3%)	105 (44.7%)			120 (51.1%)	115 (48.9%)			79 (33.6%)	156 (66.4%)		
Impairement	123	66 (53.7%)	57 (46.3%)			59 (48.0%)	64 (52.0%)			48 (39.0%)	75 (61.0%)		

### Univariate and multivariate analyses for the detection of independent prognostic factors in patients with NSCLC

The follow-up period of the entire cohort ranged from 3 to 84 months (median of 36 months). The one-, three-, and five-year overall survival (OS) rates for the whole study population were 83.5%, 51.1%, and 40.3%, respectively, with the median survival time (MST) of 48 months.

To determine the effect of pulmonary function on the prognosis, we first performed univariate analysis on the clinicopathological variables for prognosis. The results of univariate analysis for OS were showed in Table 3, of the available clinicopathological variables, age (*P* = 0.025), T stage (*P* < 0.001), lymph node status (*P* < 0.001), VC (*P* < 0.001), FVC (*P* < 0.001), FEV1 (*P* = 0.014), MVV (*P* = 0.016), NLR (*P* < 0.001), PLR (*P* = 0.033), and LMR (*P* < 0.001) were found to be significantly associated with prognosis by log-rank test.

**Table 3:** Univariate survival analysis of clinicopathological variables with regard to the OS of NSCLC patients

Variables	N	5-YSR (%)	*x^2^* value	*P* value
Age (years)			4.996	0.025
≤60	175	44.8		
>60	183	36.0		
Gender			0.077	0.782
Male	247	40.9		
Female	111	39.2		
Smoking history			0.852	0.356
Yes	244	39.3		
None	114	42.5		
Histology			0.667	0.716
Adenocarcinoma	125	39.6		
Squamous cell carcinoma	162	41.9		
Others	71	37.9		
T stage			16.805	**0.000**
T1	152	50.5		
T2	185	34.6		
T3-4	21	18.2		
Lymph node status			42.664	**0.000**
N0	219	53.6		
N+	139	19.1		
VC			12.938	**0.000**
Normal	162	50.7		
Impairement	196	31.9		
FVC			18.948	**0.000**
Normal	192	51.2		
Impairement	166	27.7		
FEV1			6.076	**0.014**
Normal	145	48.4		
Impairement	213	34.8		
FEV1/FVC			0.719	0.396
Normal	94	35.9		
Impairement	264	41.9		
PEF			0.054	0.816
Normal	78	39.5		
Impairement	280	40.6		
MEF50%			0.213	0.644
Normal	109	43.5		
Impairement	249	38.8		
DLCO			0.899	0.343
Normal	240	42.3		
Impairement	118	36.8		
MVV			5.842	**0.016**
Normal	235	45.0		
Impairement	123	31.5		
NLR			20.957	**0.000**
<2.14	196	47.7		
≥2.14	162	31.4		
PLR			4.545	**0.033**
<121.78	179	44.9		
≥121.78	179	35.8		
LMR			25.437	**0.000**
<3.06	127	27.3		
≥3.06	231	47.5		

**Figure 2:**
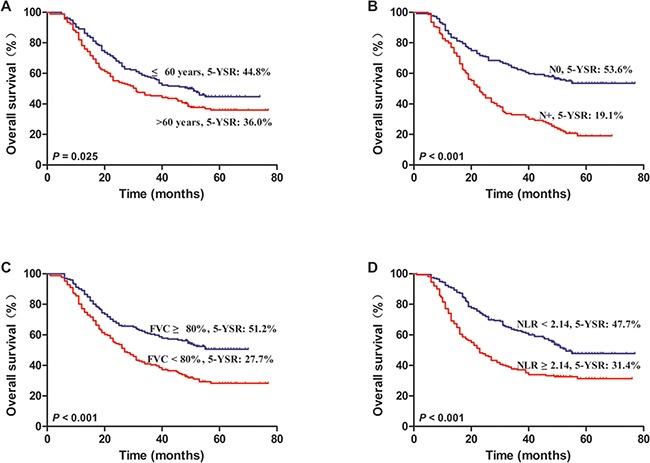
Kaplan–Meier survival curves for NSCLC patients after lung resection **A**. Patients with age ≤60 years and >60 years. **B**. Patients with and without lymph node metastasis. **C**. Patients with FVC expressed as a percent of predicted ≥80% and FVC% <80%. **D**. Patients with NLR <2.14 and NLR ≥2.14.

To determine the independent prognostic factors, we performed a multivariate analysis on the statistically significant factors in the univariate analysis. A Cox proportional hazard model showed that age (hazard ratio (HR) 1.381, 95 % CI 1.082-1.855, *P* = 0.032), lymph node status (HR 2.468, 95 % CI 1.858-3.277, *P* = 0.000), FVC (HR 1.689, 95 % CI 1.057-2.699, *P* = 0.029), and NLR (HR 1.482, 95 % CI 1.029-2.135, *P* = 0.034) were independent prognostic factors, which were significantly associated with long-term survival of patients with NSCLC (Table 4, Figure 2).

**Table 4:** Multivariable Cox proportional hazards regression analysis of OS in NSCLC patients

Variables	B	Wald	HR	95%CI	*P* value
Age	0.323	4.589	1.381	1.028∼1.855	**0.032**
T stage	0.206	2.812	1.228	0.966∼1.563	0.094
Lymph node status	0.903	38.953	2.468	1.858∼3.277	**0.000**
VC	−0.016	0.005	0.984	0.616∼1.571	0.945
FVC	0.524	4.797	1.689	1.057∼2.699	**0.029**
FEV1	0.077	0.140	1.080	0.722∼1.614	0.708
MVV	0.046	0.085	1.047	0.769∼1.426	0.770
NLR	0.394	4.471	1.482	1.029∼2.135	**0.034**
PLR	0.059	0.139	1.061	0.778∼1.447	0.709
LMR	0.283	2.331	1.327	0.923∼1.907	0.127

**Figure 3:**
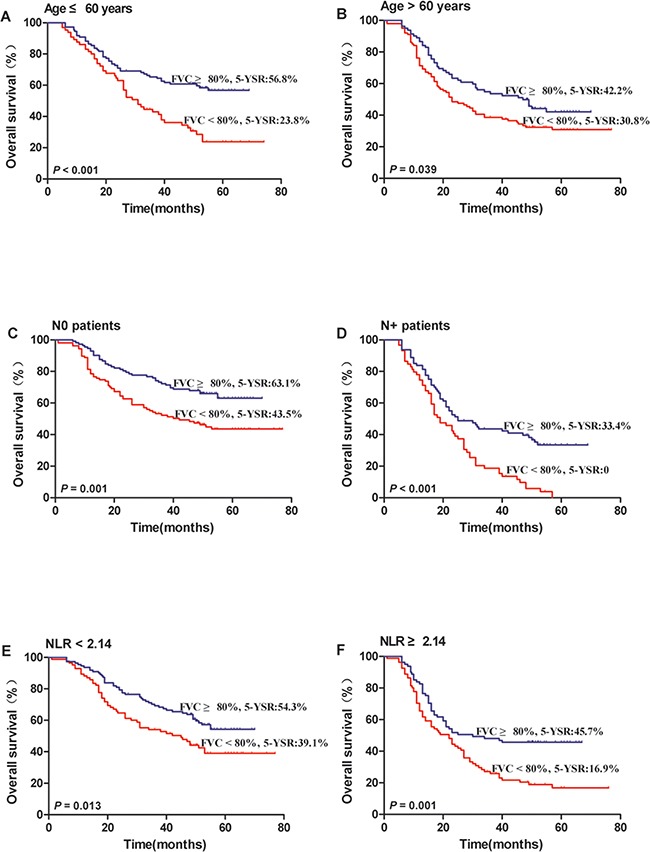
Kaplan–Meier survival analysis of the prognostic value of preoperative FVC in each subgroup All patients were stratified according to age (**A, B**), lymph node status (**C, D**) and NLR (**E, F**). (**A**) age ≤60 years subgroup, (**B**) age >60 years subgroup, (**C**) no lymph node metastasis subgroup, (**D**) positive lymph node metastasis subgroup, (**E**) NLR <2.14 subgroup, and (**F**) NLR ≥2.14 subgroup.

To further evaluate the prognostic value of FVC in different subgroups, the patients were further classified according to age (Figure 3A and 3B), lymph node status (Figure 3C and 3D) and NLR (Figure 3E and 3F). The FVC retained its prognostic value in OS prediction for all the subgroups (*P* < 0.05). Therefore, it appears that FVC may serve as a powerful prognostic factor for patients with NSCLC in different risk subgroups.

### Risk groups based on the prognostic model

**Figure 4:**
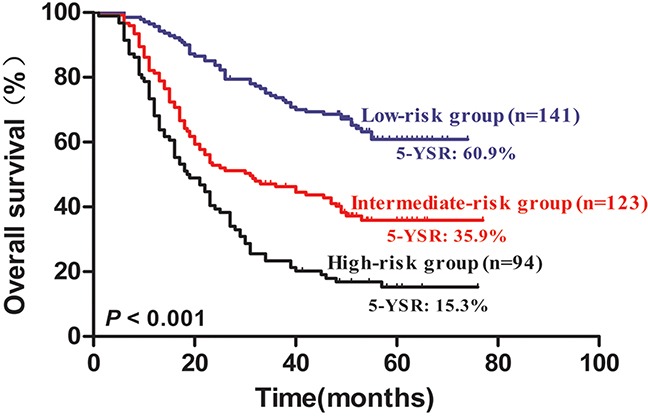
Kaplan–Meier survival curves for 358 NSCLC patients according to risk groups The five-year OS rates for low-, intermediate-, and high-risk groups were 60.9%, 35.9%, and 15.3%, respectively(*P <* 0.001).

Multivariate survival analysis identified four independent risk factors, namely, age of >60 years, positive lymph node metastasis, impaired FVC, and elevated NLR. To identify the patients with poor long-term survival, we constructed a new risk prognostic model based on the four independent prognostic factors. The patients were then assigned into the following three groups: Group I, low-risk group, patients with zero or one risk factor; Group II, intermediate-risk group, patients with two risk factors; Group III, high-risk group, patients with three or four risk factors. Among the patients, 141 were included in the low-risk group, 123 were part of the intermediate group, and 94 were included in the high-risk group. The five-year OS rates for the low-, intermediate-, and high-risk groups were 60.9%, 35.9%, and 15.3%, respectively. Based on the prognostic model, the five-year OS rates of the three groups were statistically different (*P* < 0.001) (Figure 4).

## DISCUSSION

In the present study, the associations among the preoperative pulmonary function, serum SIR biomarkers (NLR, PLR, and LMR), and prognosis were investigated in 358 patients with NSCLC. Our results first demonstrated that preoperative pulmonary function were associated with SIR biomarkers, and the FVC was simultaneously associated with the three SIR biomarkers. These results implied that increased systemic inflammatory activity may play an important role in impaired pulmonary function in NSCLC patients. Furthermore, only FVC was demonstrated to be an independent prognostic predictor in all pulmonary function parameters. Subgroup analysis results further showed that FVC retained its prognostic value in OS prediction in all the risk subgroups and may be considered as a potent prognostic factor in NSCLC patients.

For many years, SIR has been shown to be related to the presence of cancer, tumor progression, and prognosis in NSCLC [13, 14]. Inflammatory mediators and cytokines produced by tumors or a host's innate response can exert immunomodulatory effects through apoptosis inhibition, DNA damage, and angiogenesis promotion [15]. Moreover, recent data showed that elevated SIR may contribute to impaired pulmonary function not only in patients with pulmonary disease and smokers but also in healthy individuals and non-smokers [11, 12, 16, 17]. The systemic effects of cancer-related inflammation can be quantified using routinely available serum SIR biomarkers, such as NLR, PLR, and LMR. In recent, mounting data reported that objective, inexpensive and stage-independent predictors, NLR, PLR and LMR were associated with long-term survival in NSCLC [9, 10, 18, 19]. Thus, these biomarkers are becoming increasingly important in clinical applications. Meanwhile, peripheral white cell count, mononuclear cells, and lymphocytes were reported to be inversely associated with pulmonary function [12]. However, as the ratios of absolute neutrophil, lymphocyte, and monocytes counts, the relationship between SIR biomarkers and preoperative pulmonary function remains unclear. Therefore, based on these facts, we included these SIR biomarkers in our analysis and explored the relationship between preoperative pulmonary function and SIR biomarkers, respectively. Results showed that NLR, PLR, and LMR were associated with VC, FVC, FEV1, and DLCO. Notably, FVC was simultaneously related to three SIR biomarkers. However, the mechanism underlying the relationship between pulmonary function and SIR remains unclear. In the study of Yang et al. [20], elevated serum white blood cell count is demonstrated to be associated with decreased pulmonary function. McKeever et al. [12] further reported that elevated circulating granulocytes, mononuclear cells, and lymphocytes were inversely associated with FVC and FEV1. A considerable number of resident macrophages can be found in the innate protection system of lung tissues for protection against air pollution, tobacco smoking, and microorganisms. Lung protective macrophages was activated to generate various cytokines when microorganisms or particles were inhaled into lung tissue. These cytokines then regulated local inflammatory response and entered the circulation to activate the SIR [16, 21]. The SIR induced endothelial dysfunction and then contributed to pulmonary vascular filtration, lung tissue injury [22], obstructive and restrictive lung diseases, and impaired pulmonary function [16, 23]. These findings implied the possible role of inflammatory cells and cytokines in impaired pulmonary function.

In clinical practice, preoperative pulmonary functions, especially FEV1 and DLCO, are regularly used to evaluate the operability and to predict the risk of perioperative complications and mortality in thoracic surgery [3]. Percent predicted values, which adjust with gender, age, weight, height and race of patients, are thought to be more useful than absolute values [3, 24]. At present, however, previous studies have different conclusions regarding the prognostic value of pulmonary function on the long-term survival in NSCLC after lung resection. In the present study, by including more pulmonary function parameters, we explored the optimum preoperative pulmonary function parameters for prognosis prediction and subsequently observed that preoperative FVC was the only independent prognostic predictor in NSCLC. Our results were consistent with those of Guo et al. [4] and Burney et al. [25]. Although FEV1 and DLCO have been interpreted as evidence for assessing the chronic obstructive lung disease (COPD) and many cardiovascular diseases [25, 26], few studies found impaired FEV1 and DLCO were related to poor prognosis [3, 27]. Possible explanations may be improved ventilator function after resection of the obstructive disease and better perioperative care and therapy. Besides, in some studies, the pulmonary function parameters were expressed as absolute values instead of as percentage of predicted values, and additional pulmonary function parameters (such as FVC and FEV1/FVC) were not incorporated in the analysis for prognosis. Finally, different studies adopted various criteria points to stratify severity of pulmonary function. All these factors mentioned above might lead to different conclusions. On the other hand, the FVC, forced vital capacity, is mainly determined by level of physical energy, respiratory muscle function, airway resistance, and elasticity of thorax and lung tissues [4]. According to Guo et al. [4], FVC does not only reflect the pulmonary function but also the status of the entire body. The possible mechanism, to some degree, could explain why preoperative FVC was an independent prognostic predictor in NSCLC.

Furthermore, the present study extended these findings by revealing that NLR and PLR were inversely associated with pulmonary function and survival, and LMR was positively related to pulmonary function and survival. Previous studies reported that lymphocytes with targets for tumor-associated antigens could contribute to tumor suppression [28]. However, elevated neutrophils may influence the cytolytic activity of the lymphocytes, and increased NLR reflects the pro-tumor capacity of the host [29]. Circulatory mononuclear cells enter the tumor tissue and differentiate into tumor-associated macrophages (TAMs) [30]. TAMs could secrete pro-angiogenic factors to induce angiogenesis under certain conditions to promote tumor growth and metastasis [31]. Besides, previous studies revealed that increased NLR and PLR and decreased LMR were associated with poor prognosis in NSCLC [18]. Our results also demonstrated that impaired preoperative pulmonary function was associated with increased NLR and PLR, decreased LMR, and poor prognosis. Based on these data, we assumed that increased SIR led to poor prognosis in NSCLC patients with impaired preoperative pulmonary function. However, the mechanisms that connect pulmonary function, inflammatory response, and long-term prognosis of NSCLC patients require further investigation.

In the present study, age, lymph node status, preoperative FVC, and NLR were demonstrated to be the independent prognostic predictors for the long-term survival in NSCLC patients. We further construct a novel and comprehensive prognostic model based on the four independent risk factors. This prognostic model classified NSCLC patients into low-, intermediate- and high-risk groups according to the number of risk factors. For NSCLC patients in the high risk group, anti-inflammatory treatment, improvement of pulmonary function and caring, and chemotherapy may be more important for prolonging the long-term survival. As a supplement of the TNM stage in the prognostic prediction, the model of risk stratification may help clinicians to make individualized treatment protocol and improve the prognostic prediction based on the TNM stage.

The current study had some limitations. First, the study was a retrospective one in nature with a relatively small sample population, and thus leaving some groups small in the statistical analysis. Second, although we concluded that FVC was simultaneously associated with NLR, PLR, and LMR, other pulmonary function parameters didn't simultaneously related to NLR, PLR, and LMR. Third, other systemic inflammatory immune indexes, such as C-reactive protein or albumin, which were known as prognostic factors, were absent in our retrospective study. Finally, considering the the association among preoperative pulmonary function, SIR and prognosis, we indirectly speculated the association between pulmonary function and prognosis by regulating the SIR. We recognized that all results in the current study need an in-depth analysis, and to be explored in one large population.

In conclusion, our study first revealed that impaired preoperative pulmonary function was related to increased NLR and PLR and decreased LMR. Furthermore, preoperative FVC may be the optimum prognostic factor for long-term survival of patients with NSCLC independent of age, lymph node status and NLR. The introduction of a new and comprehensive prognostic model may assist clinicians to make individualized treatment protocol according to different risk groups.

## PATIENTS AND METHODS

### Patients and characteristics

From January 2007 to December 2008, a retrospective analysis was conducted on 358 histologically confirmed NSCLC patients, who underwent curative lung resection (bronchial sleeve resection, lobectomy, or pneumonectomy) and systematic lymph nodes dissection at the Tianjin Medical University Cancer Institute and Hospital. The eligibility criteria for the current study were as follows: histologically confirmed primary NSCLC, absence of distant metastasis, complete surgical resection and systematic node dissection, no clinical evidence of infection or other inflammatory conditions, without any preoperative chemotherapy or radiotherapy, with preoperative pulmonary function tests, and complete clinical and follow-up data.

Pathological diagnosis and tumor grade were reviewed and confirmed by two independent pathologists. Tumor stage and histological classification were described according to the 7th TNM staging system of the Union for International Cancer Control (UICC) for NSCLC [32]. The main adjuvant treatment for patients after operations was chemotherapy or radiotherapy, either alone or in combination according to the NSCLC guidelines [32]. Based on the medical records, the following characteristics of each patients were collected: sex, age, smoking history, surgical procedure, histological type, T stage, lymph node status, TNM stage, pulmonary function, complete blood cell counts, and other miscellaneous information. This study was approved by the Ethical Committees of the Tianjin Medical University Cancer Institute and Hospital, and all patients have written informed consent. The methods were carried out in accordance with the relevant guidelines and regulations.

### Pulmonary function tests

Pulmonary function tests were performed by one trained technician within the week prior to the surgery and reported as percent of predicted values. All patients underwent pulmonary function tests performed on a Masterscreen-PFT (Cardinal healthcare Germany 234 GmBH). The VC, FVC, FEV1, and FEV1/FVC were measured by spirometry, which was performed according to the American Thoracic Society (ATS) standards, and the lung volumes were performed utilizing body plethysmography in all patients [33]. The diffusion lung capacity for carbon monoxide corrected for alveolar volume were measured by using the carbon monoxide single-breath technique, utilizing an inhalational mixture of 0.3% carbon monoxide (CO) and 10% helium [34]. The patients were instructed to hold their breath for 10 seconds followed by a complete and consistent exhalation. The DLCO testing was repeated after 5 minutes. Two acceptable DLCO tests within 2 mL/min/mmHg were obtained, the mean value was reported for each patient. The observed values were all expressed as a percent of predicted values for a normal patient according to age, sex, height, and weight. Pulmonary function values <80% of the predicted were defined as impaired lung function [4].

### Blood cell count analysis

Preoperative venous blood samples were collected for laboratory full blood count analysis within one week before surgery. Blood cell counts were analyzed using a standard Coulter counter (Model XE2100; Sysmex Co, Kobe, Japan). The NLR was defined as the ratio between the absolute neutrophil and absolute lymphocyte counts. PLR was defined as the ratio between the absolute platelet and absolute lymphocyte counts. LMR was defined as the ratio between the absolute lymphocyte and absolute mononuclear cells counts. The optimum cut-off points of NLR, PLR, and LMR were adopted by the ROC curves for survival prediction.

### Prognostic model and risk groups

The prognostic model was established based on the risk factors according to the multivariate survival proportional hazard model. We further divided the patients into low-, intermediate- and high-risk groups according to the number of risk factors in the model. Comparisons of survival among different risk groups were analyzed by using the log-rank test.

### Follow-up

After surgery, all the patients were followed up through hospital visits, telephone or correspondence every 3–6 months. Patients alive, all death and cases lost to follow up at the last follow-up were censored. The OS was defined as the duration from the date of surgery to death or the last follow-up. The deadline of the follow-up was December 2013, and the follow-up rate was 90.2%.

### Statistical analysis

All statistical analyses were conducted using the SPSS statistical package 17.0 (SPSS, Inc, Chicago, IL). The associations between pulmonary function and clinicopathological factors and SIR were calculated using the Chi-square test, and displayed in cross-tables. An ROC curve for survival prediction was used to verify the optimum cut-off points of NLR, PLR, and LMR. The AUC was measured to estimate the diagnostic accuracy. Univariate survival analyses were estimated using the Kaplan-Meier method, and compared via the log-rank test. Multivariate analyses with Cox proportional hazards model were performed to identify the independent prognostic variables. Two-sided *P*-value < 0.05 was considered as statistically significant.

## Abbreviations

NSCLC: Non-small cell lung cancer
VC: Vital capacity
FVC: Forced vital capacity
FEV1: Forced expiratory volume in one second
PEF: Peak expiratory flow
MEF50%: Mid-expiratory flow when 50% of FVC has been expired
DLCO: Diffusion lung capacity for carbon monoxide
MVV: Maximal voluntary ventilation
SIR: Systemic inflammatory response
NLR: Neutrophil-to-lymphocyte ratio
PLR: Platelet-to-lymphocyte ratio
LMR: Lymphocyte-to-monocyte ratio
ROC: Receiver operating characteristic curve
OS: Overall survival
5-YSR: 5-year survival rate
HR: Hazard ratio
CI: Confidence interval
